# Sporotrichoid presentation of rubella–associated granulomatous dermatitis

**DOI:** 10.1016/j.jdcr.2026.01.056

**Published:** 2026-02-09

**Authors:** Jessica Padniewski, Daniel D. Miller, Eamonn Maher

**Affiliations:** Department of Dermatology, University of Minnesota, Minneapolis, Minnesota

**Keywords:** granulomas, granulomatous dermatitis, infectious dermatology, rubella–associated granulomatous dermatitis, sporotrichoid pattern, vaccination

## Introduction

Cutaneous rubella, specifically rubella–associated granulomatous dermatitis, was initially described in patients with inborn errors of immunity (IEI) in 2014 highlighting a potential link between attenuated viral exposure and granuloma formation in immunocompromised individuals.^1^^,^^2^ Since then, cases of vaccine-derived and wild-type rubella virus (RuV) granulomatous lesions have been reported in both immunocompromised and immunocompetent patients.^3^, ^4^, ^5^

Historically, wild-type RuV was the predominant cause of reported cutaneous rubella cases, however, there is a notable increase in vaccine-derived cases reported. Because this is a newly described entity and >90% of Americans have received the measles, mumps, and rubella vaccine suggesting that a large portion of the population could theoretically be at risk; however, this possibility requires further investigation to better characterize actual risk.^6^ Commercial testing is not available and this may lead to under testing and thus underdiagnosis. Given the recent description of this entity, the uncertainty regarding the population at risk, and the difficulties with diagnosis, the full spectrum of cutaneous rubella manifestations may be incompletely elucidated to date.

Many cases of rubella–associated granulomatous dermatitis are initially diagnosed as “idiopathic granulomatous lesions.”.^5^ Rubella-associated granulomas may develop 3 weeks to decades after exposure and have various presentations that can mimic numerous other conditions including sarcoidosis, foreign body granulomas, tuberculosis, and other infectious granulomas.^7^^,^^8^ We present a previously unreported presentation of rubella–associated granulomatous dermatitis progressing in a sporotrichoid pattern that improved with infliximab therapy.

## Case report

**Fig 1:**
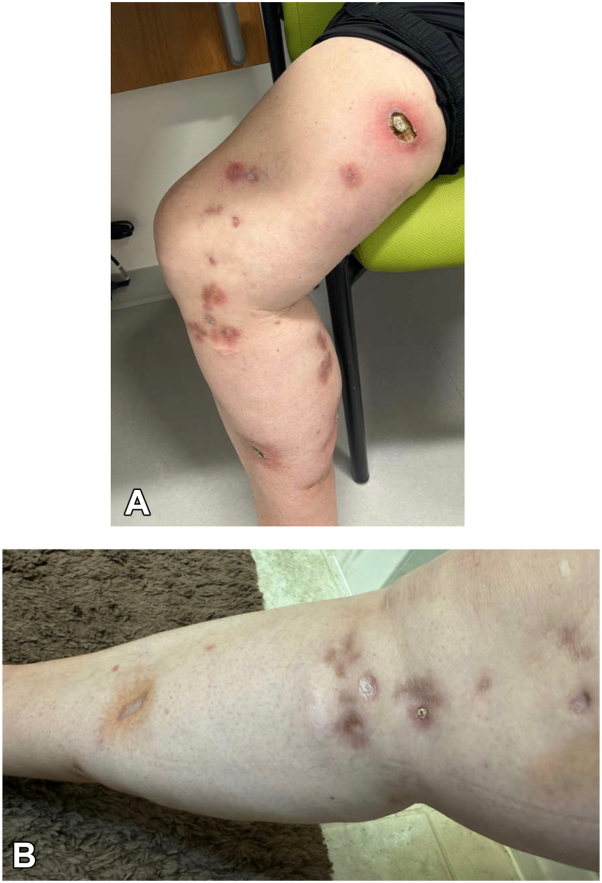
**A,** Clinical photographs of nodules demonstrating ulcerated and erythematous nodules with surrounding rim of erythema on the right medial thigh, knee, and calf with sporotrichoid pattern. **B,** Clinical photographs showing improvement after infliximab therapy.

A 56-year-old woman with a medical history of rheumatoid arthritis on abatacept and methotrexate (previously on etanercept and adalimumab) presented to dermatology clinic with ulcerated and erythematous nodules with surrounding rim of erythema on the right medial thigh, knee, and calf (Fig 1). She otherwise reported no new exposures, medications, or travel.

**Fig 2:**
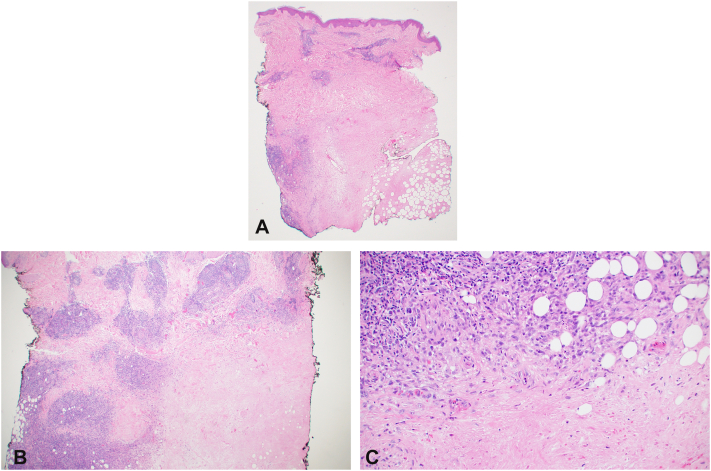
**A,** Palisaded granulomatous inflammation broadly involving the reticular dermis. **B,** Dense perivascular lymphohistiocytic infiltrate with adjacent palisading granulomas. **C,** Palisaded granulomatous inflammation with central fibrinoid necrosis and karyorrhexis. (**A-C,** Hematoxylin-eosin stain; original magnifications: **A,** ×20; **B,** ×40; **C,** ×200.)

**Fig 3:**
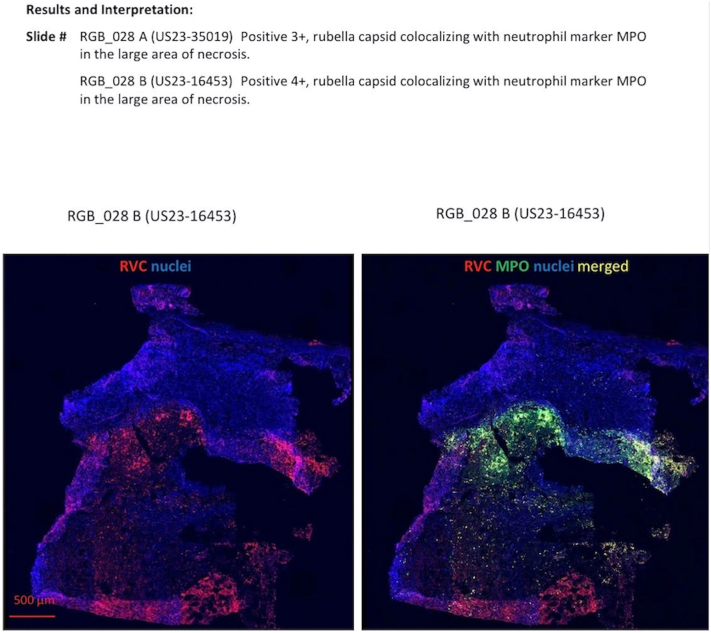
Results of immunohistochemical molecular testing amplifying rubella RNA from our patient’s skin biopsy.

Initial punch biopsy revealed palisaded granulomatous dermatitis with fibrinoid necrosis (Fig 2, *A-C*). Tissue cultures and polymerase chain reaction for fungi, bacteria, and mycobacteria were performed and were all negative for evidence of infection. She was evaluated by rheumatology and antineutrophil cytoplasmic antibodies associated vasculitis was ruled out with negative serologies. Computed tomographic angiography was performed, which demonstrated arterial patency to the areas of necrosis. She was started on dapsone and pentoxifylline, however, she failed to improve after 3 months. She underwent multiple repeat biopsies all with similar findings and ultimately she was transitioned to infliximab infusions and improved within 2 months. Given the histological features, context of immunosuppression and extensive negative work up, a tissue sample was sent to Centers for Disease Control and Prevention for RuV testing (https://www.cdc.gov/rubella/php/laboratories/rna-detection.html), which returned positive confirming the diagnosis of rubella–associated granulomatous dermatitis (Fig 3). Interestingly her skin lesions progressed in a distal-to-proximal sporotrichoid pattern (Fig 1), not previously reported per our review, and improved on antitumor necrosis factor therapy, which has been reported once per our literature review by Buchbinder et al.^3^

## Discussion

After the initial 2014 observation of RuV in cutaneous granulomas of pediatric patients with IEI, subsequent reports have documented granulomatous lesions associated with both vaccine-derived and wild-type RuV in diverse patient populations, spanning immunocompromised and immunocompetent individuals. For example, Shields et al^9^ described a case of wild-type rubella-associated granulomas in a patient with common variable immunodeficiency, in which the clinical presentation mimicked sarcoidosis or cutaneous T-cell lymphoma. Similarly, Wanat et al^10^ reported 4 cases of wild-type rubella-associated granulomas in immunocompetent patients without a history of serious systemic disease, further demonstrating the potential for RuV to induce granulomatous reactions across a spectrum of immune function.^10^ Although our patient lacked a diagnosed IEI, they had received various immunomodulatory therapies for rheumatoid arthritis over the course of several years before presentation, suggesting a state of acquired immunocompromise.

Histopathologically, cutaneous rubella granulomas are most frequently characterized by palisaded formations composed of histiocytes, lymphocytes, and giant cells. Notably, RuV antigens and RuV-infected cells exhibit distinct distribution patterns within these granulomas, correlating with the predominant cell type. These lesions can be classified into several subtypes, including nonnecrotizing macrophage-predominant, necrotizing macrophage-predominant, necrotizing neutrophil-predominant, and diffuse neutrophil inflammation intermixed with macrophages and T cells, reflecting the varied immunological responses to RuV in the skin.^8^

Rubella–associated granulomatous dermatitis frequently exhibits resistance to conventional treatments, and can result in scarring, pigmentation changes among other complications. In patients with IEI, chemotherapy and hematopoietic stem cell transplantation have demonstrated therapeutic benefit; however, these interventions are associated with significant risks and potential complications and would not be considered in patients where they are otherwise not indicated. Notably, the patient’s condition improved after treatment with infliximab, supporting the potential role of tumor necrosis factor-α inhibitors as a therapeutic option for recalcitrant cutaneous rubella granulomas.^11^ Further research is imperative to elucidate specific therapeutic targets and develop safer, more effective treatment algorithms and strategies for this challenging condition.

Rubella–associated granulomatous dermatitis, now recognized in both immunocompromised and immunocompetent individuals, arising from wild-type or vaccine-derived rubella, displays varied clinical presentations. Our case uniquely illustrates this variability, featuring a distal-to-proximal sporotrichoid pattern, a presentation not previously described in the literature, in an iatrogenically immunosuppressed patient who responded to infliximab therapy. The evolving nature of this entity implies that its full clinical spectrum is not yet fully defined.

## Conflicts of interest

None disclosed.

## References

[1] Bodemer C., Sauvage V., Mahlaoui N. (2014). Live rubella virus vaccine long-term persistence as an antigenic trigger of cutaneous granulomas in patients with primary immunodeficiency. Clin Microbiol Infect.

[2] Perelygina L., Icenogle J., Sullivan K.E. (2020). Rubella virus-associated chronic inflammation in primary immunodeficiency diseases. Curr Opin Allergy Clin Immunol.

[3] Buchbinder D., Hauck F., Albert M.H. (2019). Rubella virus-associated cutaneous granulomatous disease: a unique complication in immune-deficient patients, not limited to DNA repair disorders. J Clin Immunol.

[4] Samaran Q., Clark E., Secco L.P. (2021). Granulomatous dermatitis following measles, mumps, and rubella vaccination. Pediatr Dermatol.

[5] Zhang D., Wanat K.A., Perelygina L. (2024). Cutaneous granulomas associated with rubella virus: a clinical review. J Am Acad Dermatol.

[6] Centers for Disease Control and Prevention (CDC)/National Center for Health Statistics Immunization. https://www.cdc.gov/nchs/fastats/immunize.htm.

[7] Notarangelo L.D. (2022). Rubella virus-associated granulomas in immunocompetent adults-possible implications. JAMA Dermatol.

[8] Perelygina L., Faisthalab R., Abernathy E. (2021). Rubella virus infected macrophages and neutrophils define patterns of granulomatous inflammation in inborn and acquired errors of immunity. Front Immunol.

[9] Shields B.E., Perelygina L., Samimi S. (2021). Granulomatous dermatitis associated with rubella virus infection in an adult with immunodeficiency. JAMA Dermatol.

[10] Wanat K.A., Perelygina L., Chen M.H. (2022). Association of persistent rubella virus with idiopathic skin granulomas in clinically immunocompetent adults. JAMA Dermatol.

[11] Singh N., Raza S.H., Wanat K., Glidden N., Tirado M., Jones A. (Published online August 20, 2025). Treatment of suspected rubella virus-associated granulomatous dermatitis with adalimumab. JAMA Dermatol.

